# The Indications for the Radical Mastoid Operation with a Description of Its Technique1Read before the Buffalo Medical Club, March 19, 1902.

**Published:** 1902-06

**Authors:** W. Scott Renner

**Affiliations:** Clinical Professor of Laryngology, University of Buffalo; Member of the American Medical Association; Fellow of the American Laryngological, Rhinological and Otological Society, etc.


### BUFFALO MEDICAL JOURNAL.

VOL. XLI.—LVII. JUNE, 1902. No. 11.

### ORIGINAL COMMUNICATIONS.

### The Indications for the Radical Mastoid Operation with a Description of its Technique.[1]

By W. SCOTT RENNER, M. D., C. M.,

Clinical Professor of Laryngology, University of Buffalo; Member of the American Medical Association; Fellow of the American Laryngological, Rhinological and Otological Society, etc.

IT SEEMS but proper that some outline of the great progress made in aural surgery during the last decade should be presented at a meeting of this club. This is a task to which I do not pretend to be equal. An enumeration of the development, step by step, of each technical procedure would consume your time unnecessarily, and at the same time would prevent my dwelling upon the importance of many things which progressive aurists are doing today.

I hope, under the title I have chosen for this paper, to point out some of the radical changes which have taken place in the treatment of chronic otorrhea and its consequences. I shall not attempt to compare present methods of treatment with old ones, but shall, after pointing out the fatal results of neglecting to treat chronic otorrhea, describe the anatomy of the parts in relation to the pathology of chronic otorrhea and its sequelæ.

I shall next describe the pathology of the changes that occur in the various forms of otorrhea, which will give me an opportunity to point out the cases of otorrhea that demand the radical operation for their cure, as well as those which should be treated by simpler methods; and, finally, I will demonstrate the radical operation and tell you how it differs from the so-called "Schwartze's operation," done in acute mastoiditis; at the same time I shall point out the anatomical dangers that lurk in the region of the mastoid, and I hope incidentally to

1. Read before the Buffalo Medical Club, March 19, 1902.

describe the modifications of the operation, which may be required for the treatment of the various intracranial complications of this disease. By doing this, I hope to cover an important part of the progress made in otology in recent times, but by no means all of it.

The progress made in aural surgery during recent years is equally great, if not greater, than that made in any department of surgery. The general surgeon may say that the aurist is encroaching upon his territory. That, however, is not true, for what the aurist is doing today no one was doing a few years ago.

The aurist has to thank the general surgeon for having taught him many principles which have made it possible for him, with an accurate knowledge of the anatomy of the parts, to follow the results of suppurative otitis media into the brain, blood-sinuses and the like.

Chronic otorrhea is a disease which usually has been neglected, and many people have died unnecessarily of such neglect. Sometimes the otorrhea has been recognised as the cause of death, but in many cases, especially in children, it has not been suspected of having anything to do with the disease of which the patient died.

Statistics are often misleading; a few, however, will show how frequently this disease is the cause of death through intracranial complications, such as brain abscesses, sinus thrombosis, meningitis, and the like. In 1890 Pitt found in 9,000 post-mortems made up to that time in Guy's Hospital, 57 had died from complications of middle-ear trouble, that is 1 of every 158 cases; Barker found that of 8,028 post-mortems made in three other London hospitals in twelve years, 45 had died of such complications. These deaths, of course, have nothing to do with the operative cases. Burkner found 104 deaths among 33,017 ear cases; that is, .3 per cent.; Randall found 15 deaths in 5,000 cases of ear trouble; Bezold 7 deaths out of 325 cases of chronic otorrhea.

According to Cheval there were among 1,137 cases of middle-ear inflammation, 1 death from meningitis, 2 from brain abscesses, 2 from sinus-phlebitis and 5 from pyemia.

Schwartze found in 825 internal and middle-ear diseases in Prussia, 30 deaths from complications; that is, .35 per cent.

Barker makes the point that many of the cases do not go to special hospitals, but gives the following collective report from the University College Hospital, London, and his personal clinic.

He found that of 820 cases of acute and chronic purulent diseases, 2½ per cent. died of complications, between 1877 and 1888.

The relative frequency of the different intracranial complications have been made out from the literature on the subject in the Archives of Otology, volumes 1 to 30, and the *Zeitschrift für Ohrenheilkunde*, volumes 1 to 23, to be the following: sinus-phlebitis and pyemia were found in 41 cases; brain abscesses in 43 cases; uncomplicated meningitis in 31 cases; total, 115 cases.

Among these 115 cases there were 50 abscesses and 52 diseased sinuses. In Pitt's 9,000 cases there were otitic brain abscesses, 18; diseased sinuses, 22; otitic meningitis, 25; some of these, however, are duplicates. Of course, the number of complications can be much diminished if the cases are operated upon before more serious complications set in. Early in the trouble we find only extradural purulent inflammation, which has not yet implicated the sinuses, the pia, or the substance of the brain.

When we consider the relative frequency of the complications due to diseases of the ear or to other diseases, aurists who only see the aural brain complications naturally give the percentage due to ear diseases too high. For instance, according to Schwartze and Barr, 50 per cent. of all brain abscesses are due to aural causes. In order, however, to arrive at a more correct estimate, we must again take the statistics of general hospitals. For example, among Pitt's 9,000 post-mortems there were 56 cases of brain abscesses, the causes of which were as follows: abscesses of brain, due to purulent diseases of the ear and temporal bone, 18; abscesses of brain, due to purulent diseases of the ear and other cranial bones, 8; abscesses of brain, due to traumatic causes, 9; abscesses of brain, due to diseases of the lungs, 8; abscesses of brain, due to pyemia, 9; and 4 to unknown causes—56 in all.

Treitel found in 6,000 post-mortems made at the "Charite" in Berlin, that there were 21 cases of brain abscesses, of which 7 were of aural origin. So that from these general hospital statistics it appears that one-third of all brain abscesses are due to diseases of the ear.

Pitt found that of 44 cases of sinus-phlebitis and thrombosis, 22 were due to diseases of the ear and temporal bone; 4 were due to purulent diseases in the neighborhood of the sinuses; 3 were due to carbuncle; 7 were due to traumatic causes; 8 were due to weakening diseases. Leaving out the simple thrombosis without phlebitis, two-thirds of these cases are of aural origin.

The frequency of meningitis as a complication of middle-ear trouble is much less, for there were of all forms of meningitis among Pitt's 9,000 post-mortems, only 25 due to aural causes, of which 15 were uncomplicated, while there were 162 due to tuberculosis, and 133 due to different causes, including syphilis; that is to say, aural meningitis, compared to other forms of meningitis, is rare. The majority of those in whom intracranial complications were found in post-mortems, were between the ages of 10 and 30. A less number die from ear trouble between the first and tenth year than in either of the following decades.

Intracranial complications of chronic diseases of the temporal bone are much more common than in the acute purulent trouble of the ear. This probably is due to the fact that the chronic is of so long standing; some cases lasting from 25 to 50 years, without producing infection of the intracranial cavity.

Bergmann saw 2 cases of 80 years each who had had purulent discharge after scarlet fever since early childhood.

Since we have established the fact that chronic otorrhea is a dangerous disease because it causes intracranial complications, which will certainly produce death if not prevented by an early operation, or unless they are cured by an immediate operation as soon as they have developed, it seems necessary that we should point out the course which the purulent trouble follows from the temporal bone to the interior of the cranial cavity. The entrance of pus into the intracranial cavity is possible through 5 out of 6 walls of the tympanum, as well as through the roof of the antrum and the mastoid cells.

First, the superior wall or tegman tympani, consists partly of the petrous portion, and partly of the squamous portion of the temporal bone. In new-born children these two bones are separated by a space, which also crosses the tegmen of the antrum, through which a portion of the dura mater extends. This forms a connection between the lining of the tympanum and the intracranial cavity and contains fine branches of the middle meningeal artery, which spread out over the anterior wall of the antrum. This space closes at the fifth month of child life, and is represented later by a suture, and yet we find intracranial purulent complications much less frequently in nursing children than in adults, because in children the pus can pass out through the same fissure to the surface of the mastoid, where it forms an abscess under the soft external covering, the skin and periosteum. The thickness of the tegmen varies greatly. Sometimes it is only the thickness of paper; sometimes it has holes through it; while in other cases it is many mm. thick. The purulent inflammation of the intracranial cavity does not seem to pass through these holes. It occurs more often where there is a carious destruction of the roof of the tympanum, when it results in meningitis and brain abscesses.

The lower wall of the tympanum is, at the same time, the floor of the tympanum and roof of the bulb of the jugular vein. This wall is thin in new-born children, but depends for its thickness on the size of the jugular fossa. Where the wall is thin the bulb of the jugular can be seen through the tympanic membrane. The bulb of the jugular has been incised five times while doing a paracentesis. It is probable that phlebitis of the jugular occurs through this more frequently than is recognised, for the tympanum and the jugular fossa are seldom examined post-mortem.

Anteriorly the tympanum is separated from the carotid canal by a bony wall through which pass one of two fine canals. Through this canal passes a fold of the tympanic mucous membrane. This contains arterial vessels which pass from the carotid artery. The accompanying veins pass through it to the vein which empties into the cavernous sinus.

The inner wall of the tympanum forms the outer wall of the labyrinth. Pus may pass to the labyrinth through the membranes of the fenestræ; or infection may follow the facial canal from infection entering it in its passage through the tympanum. Or, again, infection may enter the labyrinth where the capsule of the cochlea is destroyed by caries.

The posterior wall is made up of the aditus ad antrum, so that the roof of the tympanum and that of the antrum are on the same level and support the brain. In the lower part of the antrum is found a covered canal for the facial nerve, as well as one for the semicircular canal. Infection may here also enter the facial canal along with its branches—namely, the branch to the stapedius muscle and the chordi tympani; or through defects in its wall caused by caries or pressure of a cholesteatoma. In childhood pus may be carried to the brain through an opening which enters the cranial cavity beneath the semi-circular canal.

In new-born children the antrum is a cavity the size of a cherry stone. The tympanum is the size of that of an adult.

The antrum in an adult is the size of a small bean. In a new-born child there are no other spaces besides the antrum, the mastoid being at that time but slightly developed. At the third year the mastoid cells are found, and are then similar to those found in the adult. They all open into the mastoid antrum. The farther they are from the antrum the larger they grow. Where the bone is not diseased the largest cells are found in the tip of the mastoid. They sometimes extend into the occipital bone, into the zygomatic process, and along the Eustachian tube. Compact bone is not found in healthy mastoids; some are made up of spongy bone and contain but few spaces besides the antrum. The tympanum, the antrum, and the mastoid cells are in direct atmospheric communication with each other and are lined with a continuous muco-periosteal membrane.

There are many sources of chronic purulent discharge from the ear. It may arise from the tympanic mucous membrane, from disease of its bony walls, from polypi, and granulation in its cavity, or it may arise from the mucous membrane of the antrum, and from its bony walls.

I wish to dwell on certain forms of purulent inflammation which at times require the radical operation, and to tell when and why this operation is required. We can understand these cases better after we have become acquainted with one of their most constant companions—namely, osteosclerosis of the mastoid cells, which is found associated with any purulent inflammation of the mastoid, except purulent tubercular inflammation, or purulent inflammation of the mastoid in tuberculous individuals. Osteosclerosis is due to the deposit in the mastoid, of new bone in compact layers in the walls of the cells and elsewhere, until all that remains eventually, in some cases, of the air spaces is the antrum, which is surrounded by compact bone. The antrum is also sometimes lessened in size and may be obliterated. The auditory canal may be narrowed by this process. The density of the bone depends upon the duration of the disease. Where the process is advanced, the bone is greyish-whitish in color and without bloodvessels.

Chronic purulent discharge is always accompanied by some sclerotic change in the bone; this, of course, is not great where the discharge has lasted but a short time. This sclerosis does not of itself make the disease dangerous; it often protects the sigmoid sinus. The danger is caused by an obstruction to the discharge of pus from the antrum into the auditory canal, then infection is driven through some of the preformed canals in the bone, or through caries of the walls between the antrum or attic and the cranial cavity, unless the obstruction to the discharge of pus is immediately relieved. Sclerosis, however, does not always resist the purulent process, for its walls are destroyed by it. Before opening a diseased mastoid we cannot estimate how much sclerosis we are likely to find. A long-standing purulent inflammation will naturally make us suspect sclerosis. Chronic inflammations here may be divided as follows:

1. Chronic purulent disease of the temporal bone (not associated with migration of epidermis from the tympanum into the mastoid).

2. Chronic purulent disease, associated with migration of the epidermis; that is, the so-called pseudo-cholesteatoma.

3. Congenital or true cholesteatoma, associated with purulent inflammation.

*The first of these, chronic mastoiditis*, is a disease of late childhood and early adult life which commenced in an acute otitis media five or ten years before; probably with an attack of scarlet fever or measles. Of Korner's cases of chronic mastoiditis 94 per cent. were between childhood and the 30th year, or 57 per cent. of them were between the 10th and 20th year.

Acute mastoiditis is a rapidly destructive process of the walls of the cells of the mastoid, *i. e.*, an ostitis or osteomyelitis. Chronic mastoiditis is a slow process, confined to the walls of the antrum by the surrounding sclerosis. The antrum may be enlarged through the chronic disease, its walls being made up of softened discolored bone impregnated with granulations. Fistulous openings leading to the antrum are rare in chronic mastoiditis. When they are found they pierce the cortex through the mastoid fossa or the posterior superior wall of the auditory canal, the two places through which the antrum communicates with the surface by means of bloodvessels. When fistulæ of long standing are found in either of these places, they are usually due to an acute mastoiditis, occurring at the time of the primary otitis media. The mucous membrane of the antrum is generally destroyed or impregnated with granulations. The antrum itself is frequently filled with granulation tissue, but may contain inspissated pus, clear pus, or a simple clear jelly-like substance.

The walls about are, of course, either sclerosed or partly sclerosed. When the process has extended far enough, there is left only the outer thickly sclerosed wall. The external surface is usually unchanged. The tympanic membrane is usually destroyed. The ossicles are sometimes destroyed. The tympanic cavity may be filled with granulations of polypi. Facial paralysis may occur. The caries of the roof of the tympanum and antrum may be found leading to the intracranial cavity with the consequent complications. The intracranial complications of this form of chronic mastoiditis are almost always the result of an acute exacerbation. These exacerbations occur by fits and starts, and have about the same symptomatology as an attack of acute mastoiditis—namely, fever, pain in the mastoid and tenderness on pressure. These attacks, with the rapid development of their complications, are the result of the retention of pus in the middle-ear spaces by an obstruction somewhere in the line of exit. This may occur in the aditus, the attic, the tympanum, or the external auditory canal, and may be brought about by inspissated pus, granulations or polypi.

In this class of cases we find a purulent discharge which has lasted for years. It may be very small in amount and has a very unpleasant odor. Crusts of inspissated pus, dislodged when the auditory canal is washed out, may be the only evidence of the discharge. The external auditory canal is often filled with granulations or polypi springing from the tympanum. The tympanic membrane is usually nearly gone. The hammer may be present with its handle extending, non-adherent, into the perforation, or it may be adhered to the promontory. The anvil is usually lost before the hammer. When no polypi or granulations are found the mucous membrane of the tympanum is thickened. The amount of hearing in these cases varies with the conditions of the tympanum, or the ossicles, and of the fenestræ, and the condition of the labyrinth to which inflammation may have extended through the fenestra, through caries of the capsule of the cochlea, or through a fistula of the horizontal semicircular canal in the antrum. Disease of the labyrinth is accompanied by dizziness and nystagmus.

Disease of the bone may occur without producing pain, and when pain does occur it is due to the retention of pus by obstruction to its discharge. These pains radiate to the whole side of the head. This pain should be distinguished from that due to a furuncle in the auditory canal. Pain due to retention of pus is generally associated with fever, otherwise chronic destruction of the mastoid, when not complicated, runs its course without fever. Tenderness on pressure over the mastoid is a frequent symptom of pus retention. Swelling of the external covering of the bone is rarely found except in children, and then only when the external surface of the bone is diseased, either in the mastoid fossa, or the posterior-superior wall of the auditory canal. Then we have infiltration of the skin, subperiosteal abscess and fistula through the skin, similar to that which we find in the acute mastoiditis of children, by breaking through of the pus into the digastric fossa. Bezold's mastoiditis may occur here, but much less frequently than in the acute mastoiditis. The general disturbance is not so marked as in acute mastoiditis. Cerebral symptoms such as hemicrania and loss of the power of concentration occur in chronic mastoiditis only when there is pus retention.

*Diagnosis of the Chronic Purulent Disease of the Temporal Bone.*—We will commence with the easier cases and go on to those more difficult.

1. If one finds a fistulous opening on the external surface of the mastoid, extending into the bone and associated with a chronic discharge from the middle-ear, there is a destruction of that part of the bone extending to the antrum.

2. If a fistulous opening is found in the posterior superior wall of the auditory canal, associated with a chronic discharge from the middle-ear, one must decide whether this fistula is associated with destruction in the mastoid, and whether it leads to the antrum, or whether it is only superficial and due to an erosion of the bony wall of the canal, following a furuncle. If the bone defect exists on the outer wall of the attic, the disease of the bone may be confined to this place.

3. If a scar, due to a fistula, or to a previous operation upon the mastoid is found, associated with chronic otorrhea, it is quite probable that there is still disease of the bony wall of the antrum. The diagnosis can only be confirmed by careful observation, according to the rules yet to be laid down.

4. If pain in the ear, in a case of chronic otorrhea accompanied by tenderness on pressure over the mastoid, or swelling in that region, is not due to a furuncle or retention of pus in the tympanum by some obstruction, such as polypi, it is due to a disturbance in the interior of the bone.

5. There are many cases of chronic otorrhea associated with disease of the bone in which there are none of the above-named conditions, either of the exterior of the bone, or the auditory canal, and yet the amount of bone destruction may be extensive.

The difficulty of diagnosis in these cases may be lessened by the following explanation. If the amount of pus is excessive, it cannot come from the tympanum alone. Should it come from above, it may arise from an extradural abscess or an abscess in the temporal lobe through a fistulous opening in the roof of the tympanum. Should it come from above and behind, then it must come from the antrum and mastoid. If the pus is fetid and does not lose its odor after repeated washing out with a tympanic syringe, it must come from a part not reached by the solution. If this syringing brings down regularly from above and behind masses of inspissated pus, it is certain that there is diseased bone, but if the pus loses its odor and contains no cheesy masses, it may be that there is only a purulent inflammation of the mucous membrane lining the antrum. This occurs in antra made smaller by osteosclerosis.

It is only possible in many cases, after long observation, to tell whether there is diseased bone and find out its extent and conditions, by the removal of other sources of pus formation, such as polypi, granulations, and disease of the ossicles. True and false cholesteatoma are, of course, also sources.

A few words in regard to the prognosis of cases in this class, which are not operated upon. Korner found that 94 per cent. of his cases of chronic mastoiditis operated upon were under thirty years, and yet there are many old people with chronic otorrhea; therefore, there are many who go beyond this time that seldom have mastoiditis. The prognosis in these cases cannot be held to be unfavorable. This is probably due to the extensive sclerosis which practically drives the purulent process out of the mastoid back into the tympanum.

*Prophylaxis.*—The prophylaxis of chronic mastoiditis consists of the adequate treatment of acute otitis media, and especially that due to scarlet fever, which easily becomes chronic; and further, acute mastoiditis should be thoroughly treated. The so-called "Schwartze's" operation should be perfectly done. The chronic processes easily follow a fistulous opening to the mastoid.

*Indications for the operations.*—Schwartze's operation, which is the simple exposure of the antrum with removal of the disease of the mastoid cells, has not proven itself sufficient in the chronic cases, hence the radical operation has been devised. The radical operation is indicated as soon as the diagnosis of chronic disease of the bone is established; that is, after it is established that the pus does not come from polypi, granulations, diseased ossicles or from purulent inflammation of the mucous lining of the tympanum or antrum, but from the bony walls of the tympanum or antrum.

If the diagnosis is uncertain, then the operation should be done only under the following circumstances enumerated by Korner: (1) as soon, in a case of chronic otorrhea, as there are signs of pus retention, the cause of which is not immediately discovered; (2) when there is marked bony thickness of the auditory canal, preventing examination and treatment of the discharge which may result in its retention; (3) on the commencement of conditions which favor the development of intracranial complications, such as the purulent process attacking the labyrinth or facial canal; (4) on the first signs of the commencement of an intracranial complication. Should in any uncertain case none of these conditions be found, then the case should be treated as simply an inflammation of the mucous lining. Korner says he knows of no case, either of his own or in the literature, where a simple purulent inflammation of the antrum, with no obstruction to the discharge of pus, has led to intracranial complications. Unnecessary operating, or the desire to operate on the part of the surgeon in cases which heal eventually of themselves, brings the method into discredit and does more harm eventually than the failure to operate in a case where the diagnosis is uncertain. An operation may be performed for persistent fetid otorrhea without signs of inflammation of the mastoid, and without signs of retention of pus, when it has been established by examination of the ear that the formation of pus is not confined to the tympanic cavity.

We have still to speak of the two conditions which were originally grouped together by Kummel, under the name of cholesteatoma. The first of these, pseudo-cholesteatoma, or desquamative disease of the air spaces of the temporal bone, is due to migration of the epidermis of the tympanum, under certain conditions of pus forming in the middle-ear, from the tympanum into the attic and antrum, where the epidermis collects and forms cholesteatoma-like masses. True cholesteatoma of the temporal bone is like that of the occipital and inferior maxillary bones and that of the pia mater. It is a heteroplastic growth, which has its commencement in embryonic life. It has of itself nothing to do with a purulent otitis media, but becomes frequently associated with it on account of its proximity to the tympanum and mastoid antrum.

*Otitis media desquamosa or pseudo-cholesteatoma* occurs most often after scarlet fever. This condition leads sometimes to the healing of the purulent process; at other times to the formation of cholesteatoma-like tumors in the antrum, which from pressure cause ulceration of the bone. This condition, like the simple chronic process, is accompanied by osteosclerosis, resulting likewise in the obliteration of the mastoid cells. The formation of these tumors generally occurs without general disturbances and without pain. It is only when the mass swells rapidly through an increase in the amount of pus, and when it cannot escape through the narrow passage-way, that pain and fever are produced. Small collections of epidermis in the attic may cause more marked symptoms, even making one suspect intracranial complications. In most cases the ossicles are only partly destroyed and the facial nerve is seldom disturbed.

Fistulous openings into the horizontal semicircular canal are sometimes found. This condition is followed by disease of the bony wall of the antrum and attic. Complications are produced similar to those which were described under the head of simple chronic mastoiditis. Sometimes the epidermis forms a sac-like lining to the cavity, which can be pulled out whole, while in other cases it forms a compact mass. It takes a long time before the mass is dislodged, and then only after having produced pain for some time. Where the amount of pus formation is small, it may take years before it is dislodged.

*Diagnosis of Desquamative Mastoiditis.*—When the walls of the tympanum can be viewed it is easy to see whether it is covered with this growth of epidermis or not. It is grey and glistening when dry; when moist it is of a dull grey color. It is easily distinguished from the mucous membrane. One can easily tell that there is an over-production of epidermis when dry or moist epithelium is seen behind the edges of a perforation, or when it extends into the auditory canal. The place where it makes its appearance in the canal may indicate the position of the mass in the attic or antrum. It is difficult to recognise this condition through a perforation in Schrapnell's membrane. When the flakes of epidermis cannot be seen they may be brought out by syringing.

In these desquamative cases the pus is small in amount and may dry into crusts which cover over small amounts of pus of a very disagreeable odor. To make a diagnosis of the epithelial flakes the external auditory canal should first be thoroughly cleansed, after which the tympanic syringe should be used. When the flakes of epithelium are brought down by thus syringing, they are easily recognised; only the inexperienced can mistake them for inspissated pus. Sometimes very large masses of epithelium are removed in this way, and again no epithelium is found until syringing has been repeated several times. If the space is only carpeted with a layer of epithelium, the diagnostic syringing is useless. Then the diagnosis is only made out during the course of treatment.

The prognosis for healing without operation is better in these cases than in the true cholesteatoma. The healing is rapid where the air spaces are small and the entrance to them large. If the whole mass of epidermis is discharged spontaneously, the purulent discharge often stops of itself, but it may reappear after years. Spontaneous healing may follow the breaking through of large masses of epidermis into the auditory canal. These leave large cavities, but those cases in which a large cavity is found are usually due to the discharge of a true cholesteatoma. Proper treatment through the auditory canal often heals the purulent discharge and thus brings the desquamation to a standstill. Where considerable masses are imprisoned in large cavities with a narrow opening, there is danger of wearing away of the bone and extension of the destructive process. Any obstruction to the escape of pus in these cases, whether it is due to the covering of a perforation by a mass of epidermis or to obstruction of a narrow passage by granulations or polypi, is subject to the same dangers of intracranial complications as the uncomplicated mastoiditis.

*Treatment.*—Extraction of the hammer may be performed, and this should be followed by repeated syringing of the cavity by means of a tympanic syringe with sterile water or a weak formalin solution. This should be followed by the syringing of the parts in the same manner with alcohol. Where the fistula is located in the upper part of the tympanic membrane, the granulations first should be removed, after which it should be syringed in the same manner. This may be continued as long as the patient has no bad symptoms. The ossicles may then be removed if there are signs of the condition getting worse, such as increase of pus formation, or increase of the formation of epidermis. When one has patience to follow out this method of treatment he will get frequently as lasting results as when he extracts the adherent hammer.

If the purulent discharge and the formation of epidermis do not cease after the removal of the hammer and the anvil, and the repeated syringing, there must be caries of some of the walls, and rough bone will be recognised when touched with the sound, or a granulomatous mass will be found in its stead. Sometimes the caries extends into the auditory canal and may leave a defect large enough to see the tegmen tympanum, when the case may heal spontaneously. However, it may be necessary to remove the surrounding carious parts by means of a sharp spoon. It is in this class of cases that Stacke's operation may be performed. The over-production of epidermis in the antrum stops frequently when the purulent discharge ceases. This occurs frequently after the continuous use of the tympanic syringe.

The radical operation is indicated for this condition, when the discharge and over-production of epidermis is not cured by removal of the ossicles and the continuous syringing of the tympanum, and when the same conditions occur that require the radical operation in the simple purulent discharge from the antrum and mastoid. When doing the radical operation it is only necessary to remove the lining epidermis where it lies over diseased bone.

*True cholesteatoma*, as was pointed out before, is a congenital condition which only becomes of clinical importance when it is complicated by, and infected with, the purulent inflammation; and, as the middle-ear is more or less inflamed during the acute exanthemata of childhood, whether clinically manifested or not, it is quite evident that most of these tumors which reach the air spaces of the temporal bone are apt to be infected early and thus come under the observation of a physician, while cholesteatoma of the frontal, occipital, and lower maxillary bone go unnoticed; therefore, we find them very frequently in children and young people. The majority are found clinically in the second decade. Cholesteatoma is, according to Kuhn, an epithelial, pearl-white tumor, varying in size from a bean to a hen's egg. Its surface is usually smooth, but may be uneven. It is formed of onion-like lamellæ, of polygonal epidermoidal cells, between which are often found cholesterin crystals in varying quantities. The tumor is surrounded with a fibrous membrane, which is connected with the periosteum of the cavity in which the tumor occurs.

The cholesteatomata may be found in the attic and the antrum, and the squamous portion of the bone. The bone is only destroyed by pressure when the bone is not infected by pus. Infection produces ulcerative destruction which is surrounded by an osteosclerosis. Thus, you see it is hard to say whether you have a true or false cholesteatoma. The tumor may expand the pneumatic spaces; it may extend into the tympanum; it may break through the posterior wall of the auditory canal; it may destroy the outer cortex of the mastoid, but seldom the tip. The tumor may lie for a long time under the skin and periosteum, without producing any change in the soft parts, if the growth happens to be one not infected. If infected, it will produce periosteal abscess, infiltration of the skin, and fistula. Frequently the tumor breaks through into the middle and posterior fossa of the skull, where it presses the dura and sinus out of the way. If not infected it may press on the contents of the intracranial cavity for a long time without producing symptoms; but if infected it is followed by some intracranial purulent process. The labyrinth and the facial canal may be affected in the same way. The diagnosis of such a tumor is only possible after it has broken through into the tympanum, into the auditory canal, or on the external surface. It is most often seen in the tympanum, sometimes in the auditory canal. It is seen between the granulations and the fetid pus. It looks dirty white, or appears as pearl-like pieces broken off the tumor. These pieces should be easily distinguished from the inspissated pus of chronic inflammation. If these pieces are not found, or if the tumor does not project into the canal, this condition cannot be differentiated before the operation from ordinary chronic otorrhea; and when operating you may find such a cavity from which a tumor may have disappeared.

Rupture in the auditory canal may be followed by healing, and one may find the antrum, tympanum and auditory canal united in one cavity lined with epidermis. If the tumor has no broad way of escape, the danger of rupture into the intracranial cavity is greater than in any other form of purulent disease of the middle-ear and temporal bone. Sinus thrombosis is the most common complication following such a rupture. Some cases are healed by the breaking through into the auditory canal, but most of them should have a radical operation performed as quickly as possible.

*Necrosis of the capsule of the cochlea* is often found in chronic otorrhea, usually after scarlet fever. Necrosis of other parts of the temporal bone occur frequently in scarlet fever, but as an acute process. The necrosis here, however, is a chronic process occurring several years after the acute disease. It is due to an acute exacerbation of the old purulent trouble. The signs of it are fetid pus, which is persistent, accompanied by exuberant granulations on the posterior wall of the tympanum, which recur in a few days when removed. When this wall of the tympanum is examined with a sound, roughness of the bone is found. Eventually a movable sequestrum is recognised. This may be removed in many cases through the external auditory canal, but if it cannot be dislodged in this way, a radical operation must be performed in order to remove it.

*Technique of the Operation.*—The patient should be first prepared for an aseptic operation. The side of the head should be shaven for at least two inches in every direction about the field of operation, and the skin about the auricle for some distance, as well as the auricle itself should be made thoroughly aseptic. The auditory canal must be as thoroughly cleansed as possible. When there is sufficient time this should be done on the previous day, and a moist bichloride dressing should be left on over night. Then the sterilisation of the parts should be repeated before the operation is commenced. When the patient is well under the anesthetic an incision should be made down to the bone, as close to the auricle as possible, from the tip of the mastoid to a point just above the upper attachment of the auricle. Then the periosteum should be shoved back so as to expose the whole cortex of the mastoid. This part of the operation is the same in both Schwartze's operation and in the radical operation. In Schwartze's operation the auditory canal is not interfered with. In the radical operation the lining of the auditory canal is separated from the posterior wall down to the tympanum, from which it is not generally necessary to separate it, for the tympanic membrane is usually destroyed in the class of cases for which this operation is done. Of course all bleeding must be stopped before we go farther with the operation.

We next look for the place above which we dare not remove the bone. This is the linea temporalis, which is the continuation backwards of the upper border of the zygomatic process, and is, at the same time, the upper border of the mastoid fossa, which is the external landmark of the mastoid antrum. Just here, on the edge of the external auditory canal, is the auditory spine, which was formerly used as the upper border of the field of operation, until the temporal line began to be used in its stead. The upper border of the temporal line is on a level with the lowest part of the middle fossa of the skull, which makes it necessary for us to confine ourselves to the parts below, unless we find later that there is caries of the roof of the antrum, extending to the dura mater. After the auricle is detached by placing the end of the finger in the depression between the temporal line and the posterior margin of the external auditory canal, we cover the mastoid fossa. It is in this that fistulous openings are found, which communicate with the mastoid antrum and through which infection finds its way to the surface, to produce a periosteal abscess when no fistula exists, for this part of the cortex is perforated by several small bloodvessels. After we have satisfied ourselves as to the landmarks, we commence to chisel away the cortex of the mastoid. In acute cases we can often follow a fistulous opening to the antrum, but in chronic cases we are apt to find a great deal of osteosclerosis, which makes the chiseling very difficult. In acute cases we leave the posterior wall of the auditory canal standing, and remove the soft, diseased bone as far as it extends into the mastoid cells; but, in the chronic cases, we remove the posterior walls of the auditory canal while we deepen the opening in the bone in the direction of the mastoid antrum.

The first of these procedures is known as Schwartze's operation, and is the one which is usually done in cases of acute mastoiditis. The latter is known as the radical mastoid operation, Zaufel's or the Stacke-Schwartze operation. By it the antrum, the mastoid cells, the tympanic cavity and the auditory canal are united into one cavity, and all diseased bone, whether in the antrum or tympanum or in their walls is easily found.

Some do the radical operation by first doing a regular Schwartze operation and then removing the posterior wall after they have opened the antrum. In some cases the first stroke of the chisel will expose the sigmoid curve of the transverse sinus. In these cases the sigmoid sinus lies far forward and may be only a couple of mm. from the posterior walls of the external auditory canal.

In these cases it is necessary to do a regular Stacke operation, in which you commence to chisel away the upper and back walls of the external auditory canal, commencing where the tympanum passes into the attic. The parts below are protected by a curved instrument devised by Stacke for this purpose. The antrum is opened in this way and the bone between this and the cortex of the mastoid is removed as far back, and as thoroughly, as the position of the sigmoid sinus will allow.

In many cases when removing the diseased mastoid cells, the disease of the bone will be found to extend to the sigmoid sinus which may, therefore, be exposed for a considerable distance, for all diseased bone should be thoroughly removed. The sinus may also be accidentally exposed. The sinus wall may be perfectly healthy; it may be bathed in pus, covered with granulations, or itself diseased. Where diseased it may be filled with a thrombus. This thrombus may or may not be infected; it may be solid or broken down with formation of pus. The presence of the infectious thrombus, however, is generally expected before the operation, in which case the patient has had a rigor or repeated rigors, followed by high fever with intervals in which there was no rise of temperature. In these cases there is also pain on pressure and infiltration on the posterior border of the mastoid; symptoms of brain pressure; neuritis optica; stiffness of the neck and, when the thrombus has extended to the internal jugular vein, there is pain and a feeling of resistance in front of the sterno-cleido-mastoid muscle. When the sigmoid sinus contains a thrombus, it should be opened up and the thrombus should be removed.

It is generally a good practice to ligate the internal jugular at the same time. Where the diseased bone extends to the tegmen of the tympanum or antrum, the dura must of necessity be exposed while removing all dead bone. When we reach the dura we may find it perfectly healthy; or, we may find pus with a bolster of granulation tissue between the bone and the dura. This constitutes an extra-dural abscess. All bone covering this bolster should be removed until we come to normal dura, then the bolster should be removed. The dura should be thoroughly examined for evidence of disease or fistulous openings. Should no such be found, or should there be no symptoms pointing to abscess of the brain, it is not necessary at this time to carry the operation further. Should there, however, be symptoms pointing to abscess of the brain, the external cut should be carried forward above the auricle, and the opening through the tegmen should be enlarged until the brain is exposed at the side through the squamous portion of the temporal bone. This enlargement should be made with strong forceps in preference to the chisel. Should a fistulous opening be found over the tegmen, the abscess may be evacuated without enlarging the opening in the bone; otherwise it is better to expose the brain at the side and make incisions into its substance a couple of cm. deep until the brain abscess is discovered, when the opening to it is enlarged and drained. When doing the radical operation, beside the sigmoid sinus and the dura, we have to look out for the semicircular canal on the floor of the antrum and the facial nerve, where it passes along the posterior and inferior border of the auditory canal.

In removing this part of the wall between the auditory canal and the mastoid cells, an assistant should be directed to watch for twitching of the facial muscles. Sometimes, of course, there is already paralysis of the facial nerve, and it may be necessary to remove carious bone extending to the sheath of this nerve. After completing the operation, every vestige of granulation tissue and diseased bone should be removed from the antrum and the tympanum. In this way it is often necessary to remove the cochlea which has become carious. Of course, any remains of the tympanic membrane or diseased hammer or incus should be removed. The stapes should be left in position, for it prevents spread of the disease from the tympanum into the labyrinth.

The operation is now completed by doing one of two kinds of plastic operations. When there has been disease of the external tissues, or where we have operated for cholesteatoma, true or false, it is better to defer the plastic operation for a week or more. I prefer to do the plastic operation known as Stacke's. The other operation is the one recommended by Korner, and which is also done by Jansen, of Berlin. Stacke's operation is the one which is done in Leucæ's clinic. In the Stacke operation a flap is made of the posterior cutaneous wall, by separating it from the auricle by an incision where it is attached to this organ, and a second incision at right angles to this, along the posterior superior border of the canal. This flap is laid in the inferior portion of the bony cavity, and the dressings hold it in this position.

Some prefer, as Jansen, for instance, to close up the cutaneous wound and do all the dressing through the external auditory opening, while others prefer to let the external opening close up of itself. After it is closed the dressings are also inserted through the external auditory opening. To further this external healing, while we pack the lower part of the wound, gently but firmly, in every direction, to prevent exuberant granulations, we insert the dressings very loosely as we approach the external wound. In this way the opposing surfaces of the skin approach each other very rapidly, so that we are compelled, in a very short time, to do all our dressing through the auditory opening.

Throughout the operation no solutions are used, and blood and secretions are removed by means of sterile gauze sponges. The wound is packed with iodoform gauze; the side of the head is covered with sterile gauze, a sterile bandage is applied, and the patient, unless bad symptoms occur, is allowed to go without dressing of the wound for from five to eight days. Then the wound is dressed in this way at short intervals until the external wound closes, and until the whole posterior cavity is covered with a healthy epidermis, which will take from three to four months. After this, if all diseased bone has been removed, you are not likely to be troubled with anything more than occasional granulations, which can easily be destroyed with chromic acid. There may be at times a discharge of mucus from the Eustachian tube into the cavity, which has taken the place of the external auditory canal.

361 Pearl Street.

